# Social Determinants of Cognitive Health: Studies on Physical and Social Environments and Cognition

**DOI:** 10.1093/geroni/igab046.1464

**Published:** 2021-12-17

**Authors:** Kexin Yu, Ted Ng, Patricia Heyn

## Abstract

Living environments profoundly influence the aging process. This symposium presents research on two main aspects of the living environment and their relationships with cognitive health. The living environment is broadly defined, including both physical and social aspects. The physical environment is the characteristics of the built environment, such as tripping hazard in the home, cleanness of the community streets, and presence of deserted buildings, etc. The social environment is the cohesiveness with other people living in the neighborhood. Living environments have multiple layers; the physical environments encompass both in-home and in-community domains, whereas the social environment can be categorized as domestic versus community cohesiveness. This symposium includes studies with investigation scopes spanning from the micro to mezzo levels. The first presentation scrutinizes the buffering effect of marital relationships, as a form of domestic social environments, on cognition among older adults with vision and hearing impairments. Using the NHATS dataset, the second presentation examines social isolation as a potential mediator for the association between physical, social environments and global cognitive functioning. The third presentation evaluates the impact of living environments on cognition among Canadian older adults with multimorbidity. The last presentation examines how the physical environment affects sleep quality and thus influences older adults’ cognition. All four presentations are closely linked to the overarching theme of evaluating the environmental impact on cognition and provide possible explanations mediating the association observed. This symposium contributes to advancing gerontological knowledge by offering new perspectives on the social determinants of cognitive health.

